# The association between coronary heart disease and the risk of developing colorectal polyps: insights from the UK Biobank

**DOI:** 10.3389/fonc.2025.1643394

**Published:** 2025-11-05

**Authors:** Chi Geng, Ruochong Pang, Yuqing Xia, Yong Wu, Jianhong Zhu, Yong Chen

**Affiliations:** ^1^ Department of Cardiology, The Second Affiliated Hospital of Soochow University, Suzhou, China; ^2^ Department of Interventional Radiology, The Second Affiliated Hospital of Soochow University, Suzhou, China; ^3^ Department of Gastroenterology, The Second Affiliated Hospital of Soochow University, Suzhou, China; ^4^ Department of General Surgery, The Second Affiliated Hospital of Soochow University, Suzhou, China; ^5^ Department of Endoscopy, Fudan University Shanghai Cancer Center, Shanghai, China; ^6^ Department of Oncology, Shanghai Medical College, Fudan University, Shanghai, China

**Keywords:** coronary heart disease, colorectal polyp, colorectal cancer, UK Biobank, longitudinal approach

## Abstract

**Background:**

While numerous risk factors for colorectal polyps (CRPs) have been identified, the impact of coronary heart disease (CHD) on the etiology of CRPs remains ambiguous.

**Methods:**

This investigation involved 424,023 participants from the UK Biobank, with data collected between 2006 and 2010. We utilized Cox regression analysis and subgroup analysis to ascertain risk factors associated with the development of CRPs and to examine the relationship between CRPs and CHD. Propensity score matching (PSM) was employed to address potential confounding variables.

**Results:**

Among the 424,023 individuals with a history of CHD, the prevalence of colon polyps was 5.6%, while that of rectal polyps was 2.7%. In a longitudinal study with over 12 years of follow-up, Cox regression analysis indicated that CHD constitutes an independent risk factor for the occurrence of CRPs, a conclusion that persisted after adjusting for confounding factors via PSM. Additionally, subgroup analysis revealed that, apart from diabetes mellitus (DM), higher income, moderate physical activity, a nutritious diet, and the use of lipid-lowering medications were associated with favorable outcomes for patients with CHD, as evidenced by hazard ratio (HR) values.

**Conclusions:**

This study establishes a correlation between prolonged CHD duration and an elevated risk of CRPs. In contrast, higher income, moderate physical activity, a nutritious diet, and lipid-lowering medications are protective against CRPs in CHD patients, while DM is a risk factor. These findings support more frequent endoscopic screenings for patients with CHD.

## Introduction

Colorectal cancer (CRC) ranks as the third most prevalent cancer globally, accounting for 10.2% of all tumors in terms of incidence and 9.2% of mortality rates (1). Sporadic CRC primarily develops via three distinct pathways: the classical adenoma-adenocarcinoma pathway, the *de novo* pathway, and colitis-associated colorectal cancer. Among these pathways, the classical adenoma-adenocarcinoma pathway serves as the predominant contributor to CRC (2). Colorectal polyps (CRPs), which include adenomas and serrated polyps, are significantly correlated with the incidence of CRC (2). Prior research suggests that up to 50% of adults over the age of 50 possess at least one CRP. While the majority of CRPs are benign, a subset has the potential to progress to malignancy (3). The transition from CRP to CRC typically unfolds over an extended duration, indicating that a substantial proportion of CRC cases may be preventable through the early detection and excision of these polyps (3). Studies have demonstrated that effective screening and removal of precancerous polyps can diminish the incidence of new-onset CRC by as much as 50% (3). A recent review recommended the customization of screening methodologies based on individual risk factors for polyps and CRC; for instance, colonoscopy is advised every 10 years for individuals at average risk and every 5–10 years for those classified as high risk (4). Nonetheless, a significant challenge in screening arises from the difficulty of accurately identifying CRC risk among average-risk patients, which has prompted the investigation of novel screening strategies (5). Consequently, it is imperative to identify individuals at risk for harboring CRPs.

Coronary heart disease (CHD) results from the accumulation of atherosclerotic plaque within the coronary arteries, leading to their narrowing or blockage and subsequently reducing the blood and oxygen supply to the heart (6). The global burden of CHD is considerable, with approximately 197 million individuals affected and 9.14 million deaths attributed to the disease worldwide (6). Both CHD and CRC share common risk factors, such as metabolic syndrome, suggesting a potential linkage between these two diseases. However, only a limited number of studies have explored this relationship (7–9). For example, Lee et al. reported a significantly higher prevalence of colorectal neoplasia among patients with CHD compared to those without (7–9). Similarly, a study conducted by Chan et al. found an increased incidence of colorectal neoplasia in patients exhibiting abnormal results in coronary angiography. Nevertheless, these investigations are cross-sectional in nature and do not elucidate the risk of CRP occurrence among patients with CHD.

The present study aims to investigate the association between CHD and CRPs utilizing data from the extensive population-based cohort study, the UK Biobank.

## Methods

### Study population

Data were obtained from the UK Biobank, a prospective population-based cohort study that recruited over 500,000 participants aged 40 to 69 years across the United Kingdom between 2006 and 2010 (10). At enrollment, participants provided detailed medical histories, health behavior information, underwent physical assessments, and donated biological samples. The UK Biobank received ethical approval from the relevant research ethics committees and national governance boards, and all participants provided written informed consent. Individuals who withdrew from the study or had deceased were excluded from our analysis. This investigation adhered to the Strengthening the Reporting of Observational Studies in Epidemiology (STROBE) reporting guideline. For the primary analysis, we included 424,023 participants with a history of CHD. In the longitudinal analysis, individuals with existing colon polyps (N = 2,229) or rectal polyps (N = 1,823) at baseline were excluded, resulting in the remaining participants being involved in the time-to-event analysis.

### Assessment of colorectal polyps and other diseases

Individual colorectal polyps were assessed using data from hospital admissions, primary care records, and death registries linked to the UK Biobank. The included colorectal polyps (CRPs) and their respective International Classification of Diseases (ICD)-10 codes were K36.5 (Colon polyps) and K62.1 (Rectal polyps). The follow-up for the time-to-event analysis concluded on March 1, 2023. Participants were censored at the earliest of the following events: end of follow-up, onset of new diseases, date of death, or loss to follow-up. Prevalent cardiometabolic conditions at baseline, such as Diabetes Mellitus (DM; ICD-10 codes: E10-14) and Coronary Heart Disease (CHD; ICD-10 codes: I20-22, I24, I25), were identified based on ICD-10 codes from hospital admissions and/or primary care records.

### Assessment of covariables

Details regarding the assessment of baseline covariates have been previously described (10). In summary, we collected information on age, gender, education, employment status, alcohol consumption, smoking habits, and cholesterol medication use through touchscreen questionnaires. The Townsend Deprivation Index was utilized as an indicator of area-based socioeconomic status (11). Height (in meters) and weight (in kilograms) were measured at the assessment centers, allowing us to calculate body mass index (BMI) as weight in kilograms divided by height in meters squared. Physical activity levels were evaluated utilizing the International Physical Activity Questionnaire (IPAQ), which inquires about the frequency, intensity, and duration of walking, as well as other moderate and vigorous physical activities over the preceding four weeks (refer to **Supplementary Material**). The scoring of the questionnaire adheres to a specified protocol designed to estimate the total metabolic equivalent of tasks (METs), with total weekly METs aggregated to reflect overall physical activity levels. Moderate physical activity was defined as fulfilling one of the following criteria: engaging in a minimum of 150 minutes of moderate activity per week, 75 minutes of vigorous activity per week, or 150 minutes of a combination of moderate and vigorous activity (12). A healthy diet was assessed based on the consumption of at least four out of seven commonly consumed food groups, in accordance with dietary recommendations aimed at enhancing cardiometabolic health (13).

### Statistical analyses

For the statistical analyses, participants were categorized into two groups based on their CHD status. Pearson’s chi-squared test was utilized to examine relationships among categorical variables. To address missing data, which was presumed to occur at random, numerical variable imputation was conducted five times using fully conditional specification and predictive mean matching methods, incorporating all available data as predictors. Initially, a logistic regression model was employed to assess the cross-sectional associations between CHD and C-reactive proteins (CRPs). Subsequently, multivariate logistic regression analyses were performed, with results reported as odds ratios (OR) and 95% confidence intervals (CI). Furthermore, multivariable Cox proportional hazards analyses were executed to evaluate the associations between CHD and CRPs. Statistical significance was determined by a two-tailed P-value of less than 0.05. To address any imbalance in the CHD groups, Propensity Score Matching (PSM) was implemented utilizing the MatchIt package in R software, resulting in harmonized data. The caliper value was established at 0.01, and the balance effect was assessed using the standardized mean difference (SMD), with the objective of achieving an SMD of less than 0.1 for optimal balance (14). The methodological steps were as follows: first, propensity scores for each patient were computed employing a multivariate logistic regression model. Subsequently, patients were matched at a 1:1 ratio between the two groups. Differences across all variables were analyzed and reported based on the SMD values. Finally, correlation analysis was conducted through univariate regression analysis. All statistical analyses were performed using R software (version 4.3.2, StataCorp LLC, College Station, Texas), with results deemed statistically significant when the P-value was below 0.05. A cumulative occurrence curve was utilized to evaluate the incidence of polyps among CHD patients throughout the follow-up period.

## Results

### Basic information of study

A total of 424,023 participants were included in the study, comprising 406,054 individuals without CHD and 17,969 individuals with CHD (**Table 1**). CHD patients exhibited a greater likelihood of being female and had a higher average age compared to individuals without CHD (P<0.05). Ethnic backgrounds and levels of college education were similarly distributed between the two groups. The employment rate was lower among CHD patients than their counterparts without CHD, with a significantly higher percentage of CHD patients reporting an annual income of less than £18,000 (32.2% vs. 17.3%, P<0.001). Alcohol consumption was marginally more prevalent among CHD patients (94.3% vs. 95.4%). As anticipated, the frequency of moderate physical activity and adherence to a healthy diet were significantly lower in CHD patients compared to those without CHD (P<0.001).

**Table 1:** The basic information based on the status of CHD.

N	Overall	CHD (No)	CHD (Yes)	P value
	424023	406054	17969	
Sex, Female (%)	193068 (45.5)	184686 (45.5)	8382 (46.6)	0.002
age (mean (SD))	56.51 (8.09)	56.50 (8.10)	56.67 (8.06)	0.006
ethnic (%)				0.411
White	383316 (90.4)	367027 (90.4)	16289 (90.7)	
Mixed	38407 (9.1)	36828 (9.1)	1579 (8.8)	
Asian or Asian British	2300 (0.5)	2199 (0.5)	101 (0.6)	
University or college educational level, N (%)	287746 (67.9)	275497 (67.8)	12249 (68.2)	0.373
employment (%)				0.038
Worked	245925 (58.0)	235681 (58.0)	10244 (57.0)	
Retired	140365 (33.1)	134310 (33.1)	6055 (33.7)	
Unemployed	32943 (7.8)	31485 (7.8)	1458 (8.1)	
None of the above	4790 (1.1)	4578 (1.1)	212 (1.2)	
Income, N (%), pounds/year				<0.001
Unknown	59093 (14.0)	55940 (13.8)	3153 (17.6)	
Less than 18,000	76052 (17.9)	70259 (17.3)	5793 (32.2)	
18,000 to 30,999	91218 (21.5)	87041 (21.4)	4177 (23.2)	
31,000 to 51,999	98124 (23.1)	95240 (23.5)	2884 (16.0)	
52,000 to 100,000	78483 (18.5)	76886 (18.9)	1597 (8.9)	
>100,000	21053 (5.0)	20688 (5.1)	365 (2.0)	
Townsend deprivation index, mean (SD)	-1.36 (3.05)	-1.39 (3.04)	-0.81 (3.32)	<0.001
Smoking status, N (%)				0.237
Never	231186 (54.5)	221475 (54.5)	9711 (54.0)	
Previous	145783 (34.4)	139546 (34.4)	6237 (34.7)	
Current	44615 (10.5)	42714 (10.5)	1901 (10.6)	
Unknown	2439 (0.6)	2319 (0.6)	120 (0.7)	
Alcohol status, N (%)				<0.001
Never	18413 (4.3)	17284 (4.3)	1129 (6.3)	
Previous	13815 (3.3)	12789 (3.1)	1026 (5.7)	
Current	390612 (92.1)	374872 (92.3)	15740 (87.6)	
Unknown	1183 (0.3)	1109 (0.3)	74 (0.4)	
Moderate activity, yes, N (%)	270584 (63.8)	259839 (64.0)	10745 (59.8)	<0.001
Healthy diet, yes, N (%)	86999 (20.5)	83290 (20.5)	3709 (20.6)	0.682
waist circumference, mean (SD), mm	90.29 (13.48)	90.28 (13.48)	90.59 (13.52)	0.002
Weight, mean (SD), kg	78.05 (15.94)	78.03 (15.94)	78.43 (16.05)	0.001
Body mass index, mean (SD), kg/m2	27.43 (4.81)	27.43 (4.80)	27.54 (4.83)	0.002
glucose, mean (SD), mmol/L	5.12 (1.24)	5.12 (1.24)	5.12 (1.20)	0.762
HbA1c, mean (SD), mmol/L	36.12 (6.74)	36.12 (6.74)	36.18 (6.88)	0.202
C-reactive protein, mean (SD), mg/L	2.60 (4.35)	2.60 (4.37)	2.53 (3.94)	0.028
Triglycerides, mean (SD), mmol/L	1.75 (1.03)	1.75 (1.03)	1.77 (1.03)	0.011
High-density lipoprotein cholesterol, mean (SD), mmol/L	1.45 (0.38)	1.45 (0.38)	1.44 (0.38)	0.001
Low-density lipoprotein cholesterol, mean (SD), mmol/L	3.56 (0.87)	3.56 (0.87)	3.54 (0.88)	0.002
No-lipid lowering medication, N (%)	396942 (93.6)	383156 (94.4)	13786 (76.7)	<0.001
Diabetes mellitus				<0.001
None	391378 (92.3)	378350 (93.2)	13028 (72.5)	
Yes (during follow-up)	15737 (3.7)	13716 (3.4)	2021 (11.2)	
Yes (at basis)	16908 (4.0)	13988 (3.4)	2920 (16.3)	
Polyp of colon	23857 (5.6)	22041 (5.4)	1816 (10.1)	<0.001
Time K63.5 (mean (SD)), year	13.69 (2.15)	13.71 (2.12)	13.30 (2.87)	<0.001
Polyp of rectum	11260 (2.7)	10442 (2.6)	818 (4.6)	<0.001
Time K62.1 (mean (SD)), year	13.87 (1.87)	13.88 (1.83)	13.64 (2.54)	<0.001
Uric acid, mean (SD), μmol/L	309.08 (80.38)	309.07 (80.37)	309.39 (80.70)	0.602
Vitamin D, mean (SD), nmol/L	48.58 (21.06)	48.58 (21.05)	48.63 (21.21)	0.753

“time K62.1” represents the time-to-event for the diagnosis of a rectal polyp (ICD-10 code K62.1), and “time K63.5” represents the time-to-event for the diagnosis of a colon polyp (ICD-10 code K63.5).

Measurements including waist circumference, weight, body mass index (BMI), triglyceride levels, blood pressure, and HbA1c levels were significantly elevated in patients with CHD, whereas high-density lipoprotein levels were reduced, suggesting a propensity for metabolic dysregulation within this cohort. As anticipated, a notably smaller proportion of CHD patients received prescriptions for lipid-lowering medications compared to their non-CHD counterparts (76.7% vs. 94.4%, P < 0.001). The prevalence of diabetes mellitus (DM) was also considerably higher among CHD patients (11.2% vs. 3.4%, P < 0.001). The incidence rates of colon and rectal polyps in CHD patients were 10.1% and 4.6%, respectively, in contrast to 5.4% and 2.6% in non-CHD patients (P < 0.001 for both). Furthermore, the average duration of follow-up for patients without CHD was significantly longer than that for CHD patients concerning both colon and rectal polyps (P < 0.05).

### Associations of CHD with incident CRPs

**Table 2** and **Supplementary Table 1** delineate the associations between all variables and CRPs within the overall population. In terms of continuous variables such as age, sex, BMI, weight, and serum biomarkers, no significant differences were observed between patients with colon polyps and those without, with the exception of the Townsend Deprivation Index. For categorical variables, no significant differences in sex, ethnicity, education level, employment status, or smoking habits were detected. The results indicated that individuals with higher incomes exhibited a lower prevalence of CRPs (P < 0.001), while a greater proportion of alcohol consumers was noted among patients with CRPs. Participation in moderate physical activity, adherence to a healthy diet, and the use of lipid-lowering medications were positively correlated with the incidence of CRPs. Notably, the emergence of both CHD and DM was significantly associated with CRPs.

**Table 2:** The basic information based on the status of colon polyps.

N	Overall	Colon polyps (No)	Colon polyps (Yes)	P value
	421794	400166	21628	
Sex, Female (%)	192058 (45.5)	182220 (45.5)	9838 (45.5)	0.894
age (mean (SD))	56.51 (8.09)	56.50 (8.09)	56.55 (8.08)	0.44
ethnic (%)				0.441
White	381296 (90.4)	361696 (90.4)	19600 (90.6)	
Mixed	38207 (9.1)	36288 (9.1)	1919 (8.9)	
Asian or Asian British	2291 (0.5)	2182 (0.5)	109 (0.5)	
University or college educational level, N (%)	286239 (67.9)	271656 (67.9)	14583 (67.4)	0.161
employment (%)				0.658
Worked	244668 (58.0)	232177 (58.0)	12491 (57.8)	
Retired	139590 (33.1)	132408 (33.1)	7182 (33.2)	
Unemployed	32766 (7.8)	31047 (7.8)	1719 (7.9)	
None of the above	4770 (1.1)	4534 (1.1)	236 (1.1)	
Income, N (%), pounds/year				<0.001
Unknown	58760 (14.0)	55493 (13.8)	3267 (15.1)	
Less than 18,000	75476 (17.9)	70859 (17.7)	4617 (21.3)	
18,000 to 30,999	90701 (21.5)	85730 (21.4)	4971 (23.0)	
31,000 to 51,999	97671 (23.2)	93056 (23.3)	4615 (21.3)	
52,000 to 100,000	78206 (18.5)	74878 (18.7)	3328 (15.4)	
>100,000	20980 (5.0)	20150 (5.0)	830 (3.8)	
Townsend deprivation index, mean (SD)	-1.36 (3.05)	-1.37 (3.04)	-1.19 (3.13)	<0.001
Smoking status, N (%)				0.326
Never	229963 (54.5)	218272 (54.5)	11691 (54.1)	
Previous	145029 (34.4)	137476 (34.4)	7553 (34.9)	
Current	44375 (10.5)	42107 (10.5)	2268 (10.5)	
Unknown	2427 (0.6)	2311 (0.6)	116 (0.5)	
Alcohol status, N (%)				<0.001
Never	18328 (4.3)	17543 (4.4)	785 (3.6)	
Previous	13711 (3.3)	12907 (3.2)	804 (3.7)	
Current	388580 (92.1)	368606 (92.1)	19974 (92.4)	
Unknown	1175 (0.3)	1110 (0.3)	65 (0.3)	
Moderate activity, yes, N (%)	269205 (63.8)	255882 (63.9)	13323 (61.6)	<0.001
Healthy diet, yes, N (%)	86598 (20.5)	82539 (20.6)	4059 (18.8)	<0.001
waist circumference, mean (SD), mm	90.29 (13.48)	90.28 (13.47)	90.45 (13.55)	0.069
Weight, mean (SD), kg	78.05 (15.94)	78.04 (15.94)	78.21 (16.02)	0.141
Body mass index, mean (SD), kg/m2	27.43 (4.81)	27.43 (4.81)	27.48 (4.81)	0.172
glucose, mean (SD), mmol/L	5.12 (1.24)	5.12 (1.24)	5.13 (1.21)	0.676
HbA1c, mean (SD), mmol/L	36.12 (6.74)	36.12 (6.75)	36.13 (6.54)	0.81
C-reactive protein, mean (SD), mg/L	2.60 (4.35)	2.60 (4.35)	2.59 (4.31)	0.77
Triglycerides, mean (SD), mmol/L	1.75 (1.03)	1.75 (1.03)	1.75 (1.03)	0.899
High-density lipoprotein cholesterol, mean (SD), mmol/L	1.45 (0.38)	1.45 (0.38)	1.45 (0.38)	0.694
Low-density lipoprotein cholesterol, mean (SD), mmol/L	3.56 (0.87)	3.56 (0.87)	3.55 (0.87)	0.419
No-lipid lowering medication, N (%)	394929 (93.6)	375085 (93.7)	19844 (91.8)	<0.001
CHD	17764 (4.2)	16153 (4.0)	1611 (7.4)	<0.001
Diabetes mellitus				<0.001
None	389466 (92.3)	370862 (92.7)	18604 (86.0)	
Yes (during follow-up)	15568 (3.7)	13981 (3.5)	1587 (7.3)	
Yes (at basis)	16760 (4.0)	15323 (3.8)	1437 (6.6)	
Uric acid, mean (SD), μmol/L	309.07 (80.38)	309.05 (80.35)	309.60 (81.05)	0.327
Vitamin D, mean (SD), nmol/L	48.58 (21.06)	48.59 (21.06)	48.44 (21.02)	0.304

### Cumulative occurrence curves

**Figure 1:**
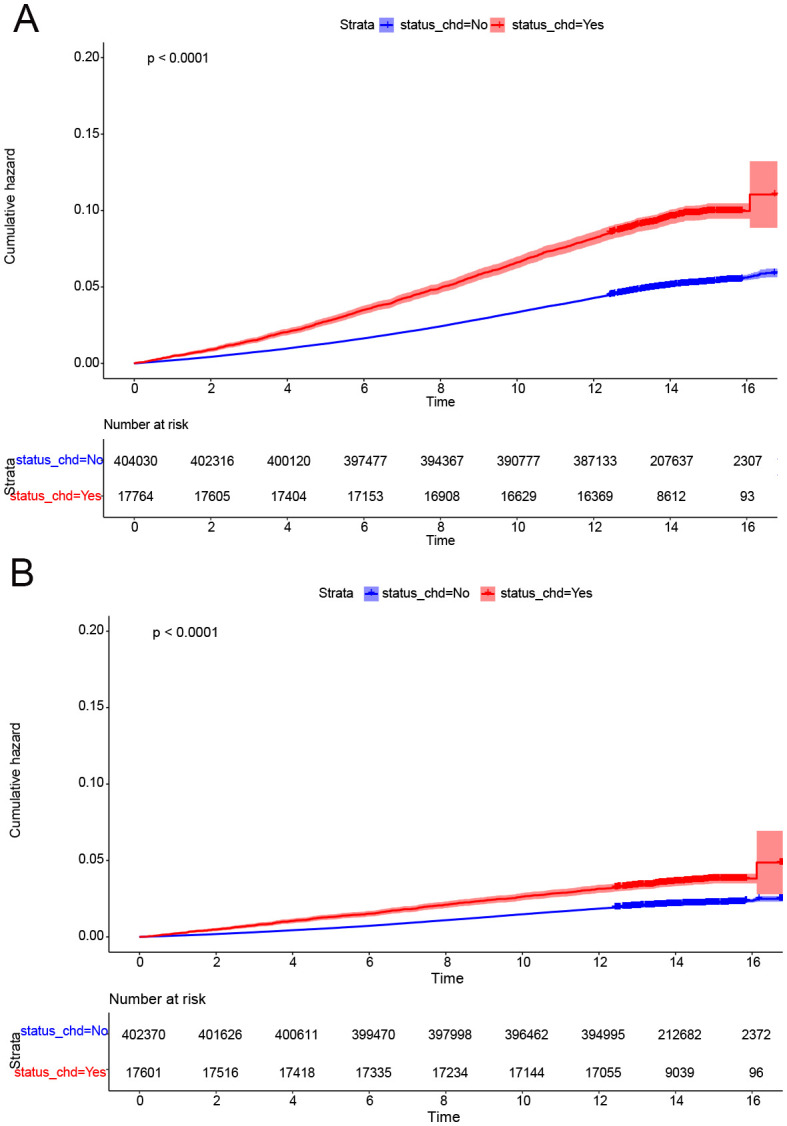
CDH was a risk factor for onset of CRPs. **(A)** The cumulative incidence rate of colon polyp in patients with or without CHD. **(B)** The cumulative incidence rate of rectal polyp in patients with or without CHD.

**Figure 2:**
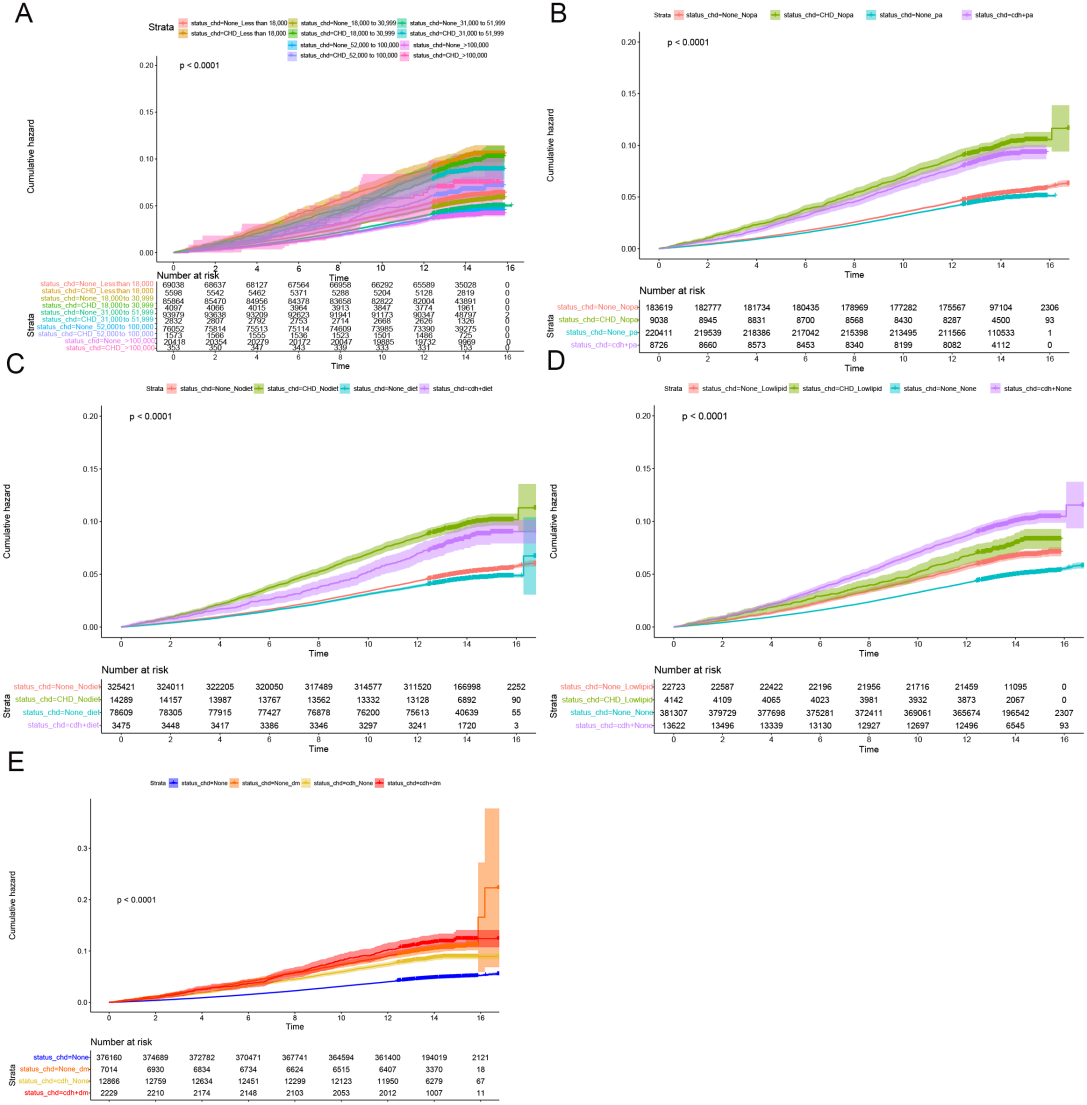
Subgroup longitudinal analysis to assess the association of CHD with colon polyps. **(A)** Grouping from top to bottom: patients with an income of less than 18000 and without CHD, patients with an income of less than 18000 and with CHD, patients with an income of 18000 to 30999 and without CHD, patients with an income of 18000 to 30999 and with CHD, patients with an income of 31000 to 51999 and without CHD, patients with an income of 31000 to 51999 and with CHD, patients with an income of 52000 to 100000 and without CHD, patients with an income of 52000 to 100000 and with CHD, patients with an income of > 100000 and without CHD, patients with an income of > 100000 and with CHD. **(B)** Grouping from top to bottom: patients without physical activity and without CHD, patients without physical activity and with CHD, patients with physical activity and without CHD, patients with physical activity and with CHD. **(C)** Grouping from top to bottom: patients without healthy diet and without CHD, patients without healthy diet and with CHD, patients without healthy diet and with CHD, patients with healthy diet and with CHD. **(D)** Grouping from top to bottom: patients with lowering lipid medication and without CHD, patients with lowering lipid medication and with CHD, patients without lowering lipid medication and without CHD, patients without lowering lipid medication and with CHD. **(E)** Grouping from top to bottom: patients without DM and without CHD, patients without DM and with CHD, patients with DM and with CHD, patients with DM and with CHD.

**Figure 3:**
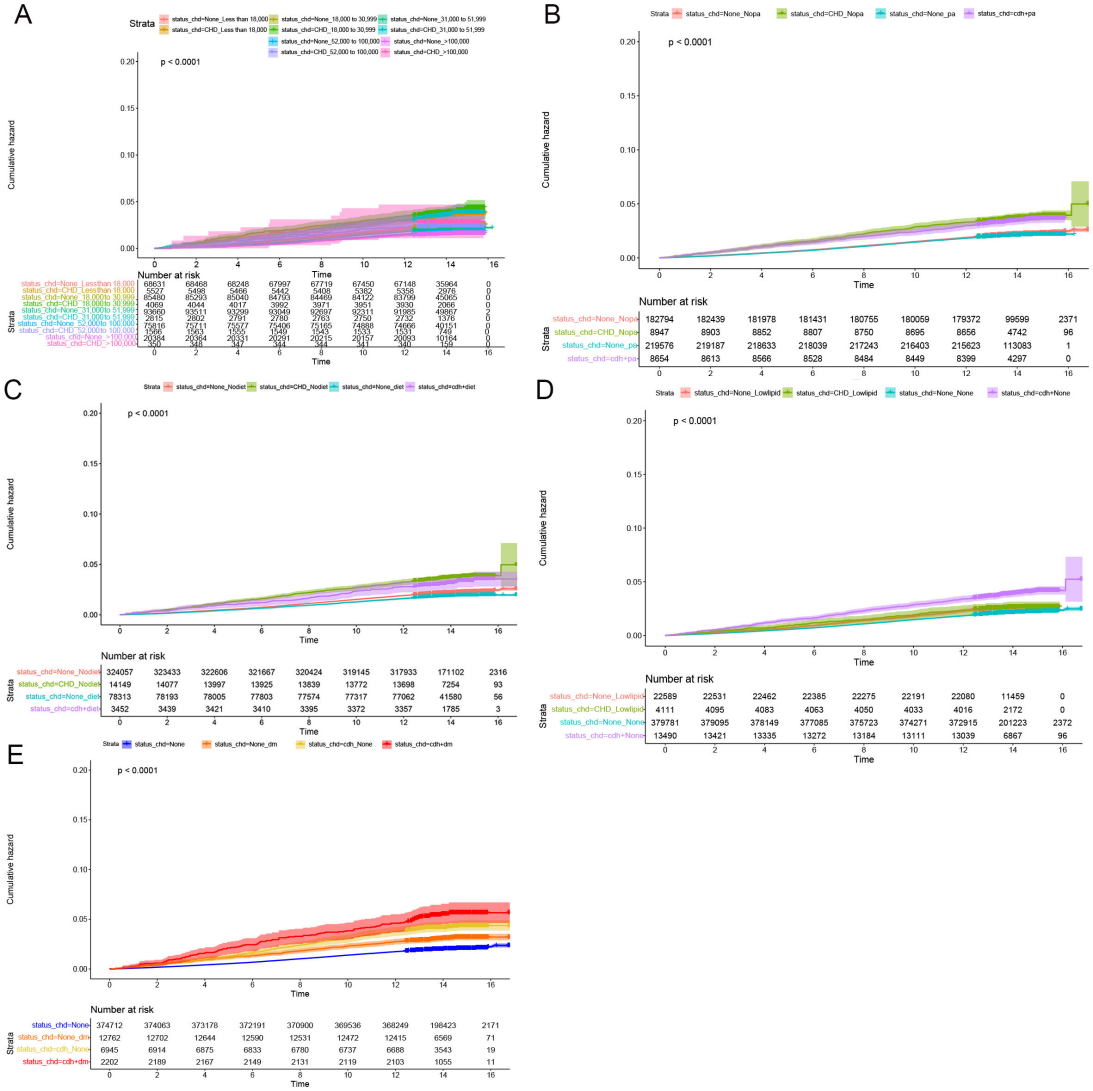
Subgroup longitudinal analysis to assess the association of CHD with rectal polyps. **(A)** Grouping from top to bottom: patients with an income of less than 18000 and without CHD, patients with an income of less than 18000 and with CHD, patients with an income of 18000 to 30999 and without CHD, patients with an income of 18000 to 30999 and with CHD, patients with an income of 31000 to 51999 and without CHD, patients with an income of 31000 to 51999 and with CHD, patients with an income of 52000 to 100000 and without CHD, patients with an income of 52000 to 100000 and with CHD, patients with an income of > 100000 and without CHD, patients with an income of > 100000 and with CHD. **(B)** Grouping from top to bottom: patients without physical activity and without CHD, patients without physical activity and with CHD, patients with physical activity and without CHD, patients with physical activity and with CHD. **(C)** Grouping from top to bottom: patients without healthy diet and without CHD, patients without healthy diet and with CHD, patients without healthy diet and with CHD, patients with healthy diet and with CHD. **(D)** Grouping from top to bottom: patients with lowering lipid medication and without CHD, patients with lowering lipid medication and with CHD, patients without lowering lipid medication and without CHD, patients without lowering lipid medication and with CHD. **(E)** Grouping from top to bottom: patients without DM and without CHD, patients without DM and with CHD, patients with DM and with CHD, patients with DM and with CHD.

Cumulative occurrence curves clearly illustrated the influence of CHD on the incidence of CRPs. **Figure 1** demonstrates that patients with CHD experienced a markedly higher cumulative hazard of developing CRPs in comparison to those without CHD (P < 0.001). A subsequent subgroup analysis (**Table 3**, **Supplementary Table 2**) revealed that across different income groups, CHD patients displayed an elevated risk of CRP onset. In alignment with the findings of the cross-sectional analysis, higher income levels were linked to a diminished risk of CRP development in both CHD and non-CHD patients, as evidenced by decreasing hazard ratios (HR) corresponding to increasing income levels (P < 0.001). Additionally, the cumulative occurrence curves indicated that elevated income levels effectively mitigated the onset of CRPs (**Figures 2A**, **3A**).

**Table 3:** Longitudinal association of CHD with risk of colon polyp based on the group of different categorical variables.

Dependent: colon polyp	Clinical variables	HR (95%CI, P value)	
		CHD (No)	CHD (Yes)
Income	Less than 18,000	Reference	1.71 (1.55-1.86, p<0.001)
	18,000 to 30,999	0.91 (0.86-0.944, p<0.001)	1.61 (1.44-1.78, p<0.001)
	31,000 to 51,999	0.78 (0.75-0.81, p<0.001)	1.45 (1.27-1.65, p<0.001)
	52,000 to 100,000	0.71 (0.68-0.75, p<0.001)	1.11 (0.91-1.35, p=0.293)
	>100,000	0.66 (0.62-0.72, p<0.001)	1.23 (0.84-1.84, p=0.287)
Moderate activity	No	Reference	1.87 (1.75-2.01, p<0.001)
	Yes	0.90 (0.88-0.93, p<0.001)	1.68 (1.56-1.81, p<0.001)
Healthy diet	No	Reference	1.88 (1.78-1.99, p<0.001)
	Yes	0.88 (0.84-0.91, p<0.001)	1.62 (1.44-1.83, p<0.001)
medication	Lowing lipid	Reference	1.18 (1.04-1.33, p=0.007)
	Other	0.74 (0.70-0.78, p<0.001)	1.49 (1.38-1.61, p<0.001)
Diabetes mellitus	No	Reference	1.81 (1.70-1.93, p<0.001)
	Yes	2.21 (2.04-2.3, p<0.001)	2.45 (2.17-2.79, p<0.001)

Furthermore, in subgroup analyses examining moderate physical activity and adherence to a healthy diet, CHD continued to be a significant contributor to the incidence of C-reactive protein (CRP), with hazard ratio (HR) values exceeding 1 (**Table 3**, **Supplementary Table 2**). These findings were further corroborated by the cumulative occurrence curve plots (**Figures 2B, C**, **3B, C**), which demonstrated that both moderate physical activity and a healthy diet were associated with a reduced risk of CRP onset in patients with CHD. Additionally, the utilization of lipid-lowering medication was linked to a diminished risk of CRP occurrence within this population, whereas a baseline diagnosis of DM was associated with an increased risk of CRP onset, as indicated by HR values and cumulative occurrence curve representations (**Table 3**, **Supplementary Table 2**; **Figures 2D, E**, **3D, E**). The associations highlighting CHD as a risk factor for CRP onset remained consistent and statistically significant across various subgroup analyses.

### Propensity scores matching analysis

**Figure 4:**
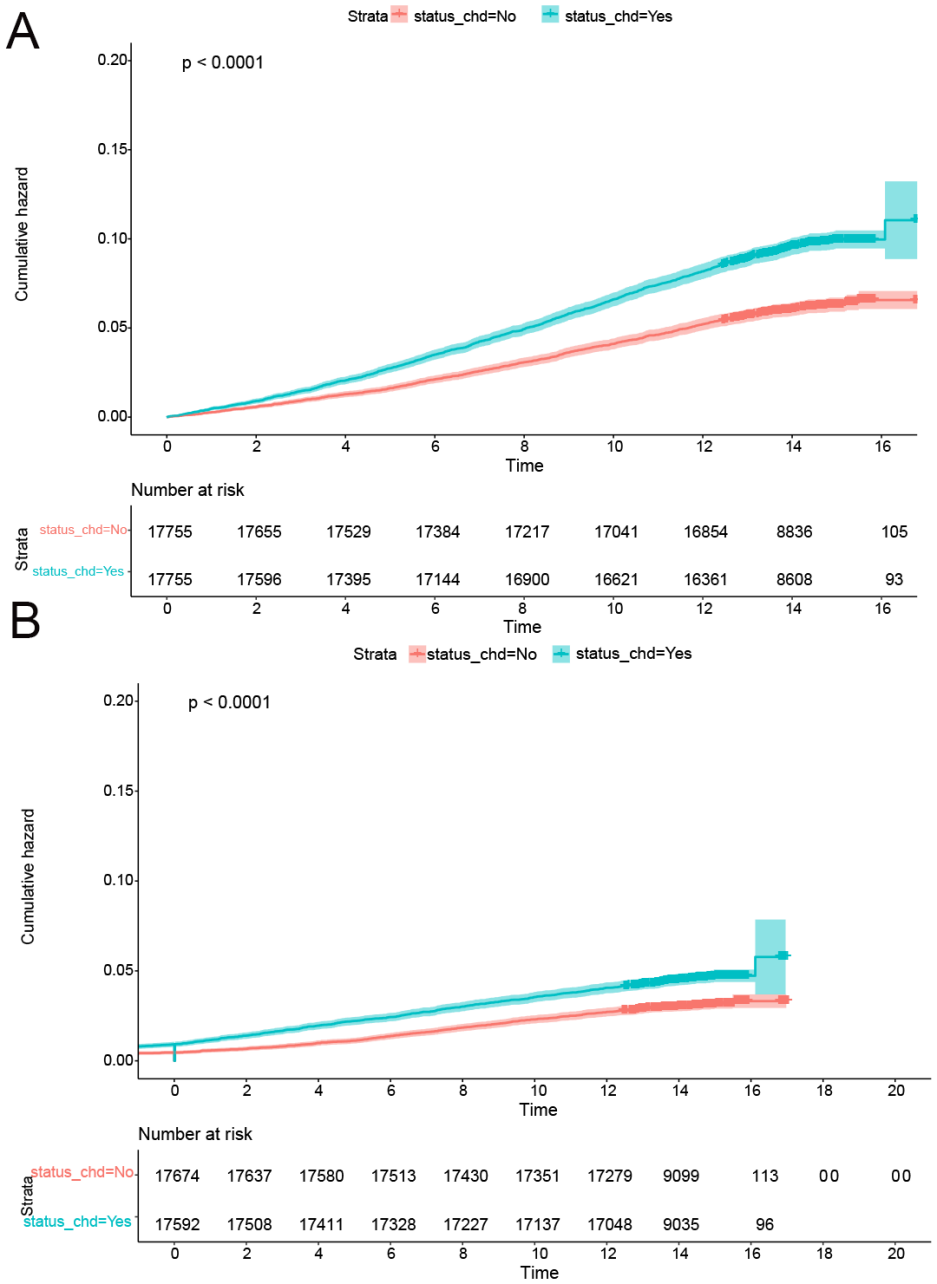
CDH was a risk factor for onset of CRPs after PSM. **(A)** The cumulative incidence rate of colon polyp in patients with or without CHD. **(B)** The cumulative incidence rate of rectal polyp in patients with or without CHD.

Following the implementation of propensity score matching, the standardized mean differences (SMD) for all covariates were below 0.1, signifying successful balance between the CHD and non-CHD groups (**Table 4**). Subsequently, both univariate logistic regression and Cox proportional hazards analysis confirmed that CHD constituted a significant risk factor for the onset of CRP. The results demonstrated a robust association between CHD and the risk of CRP development, thereby reinforcing this relationship through comprehensive statistical evaluations (**Table 5**, **Figure 4**).

**Table 4:** The basic information and SMD value based on the status of CHD after PSM.

N	Overall	CHD (No)	CHD (Yes)	SMD value
	35510	17755	17755	
Sex, Female (%)	16668 (46.9)	8377 (47.2)	8291 (46.7)	0.01
age (mean (SD))	56.68 (8.07)	56.69 (8.07)	56.67 (8.06)	0.002
ethnic (%)				0.01
White	32185 (90.6)	16087 (90.6)	16098 (90.7)	
Mixed	3139 (8.8)	1581 (8.9)	1558 (8.8)	
Asian or Asian British	186 (0.5)	87 (0.5)	99 (0.6)	
University or college educational level, N (%)	24206 (68.2)	12107 (68.2)	12099 (68.1)	0.001
employment (%)				0.019
Worked	20343 (57.3)	10217 (57.5)	10126 (57.0)	
Retired	11908 (33.5)	5930 (33.4)	5978 (33.7)	
Unemployed	2820 (7.9)	1377 (7.8)	1443 (8.1)	
None of the above	439 (1.2)	231 (1.3)	208 (1.2)	
Income, N (%), pounds/year				0.215
Unknown	6978 (19.7)	3870 (21.8)	3108 (17.5)	
Less than 18,000	9957 (28.0)	4253 (24.0)	5704 (32.1)	
18,000 to 30,999	8044 (22.7)	3902 (22.0)	4142 (23.3)	
31,000 to 51,999	6067 (17.1)	3215 (18.1)	2852 (16.1)	
52,000 to 100,000	3640 (10.3)	2053 (11.6)	1587 (8.9)	
>100,000	824 (2.3)	462 (2.6)	362 (2.0)	
Townsend deprivation index, mean (SD)	-0.78 (3.30)	-0.74 (3.29)	-0.82 (3.31)	0.025
Smoking status, N (%)				0.012
Never	19255 (54.2)	9653 (54.4)	9602 (54.1)	
Previous	12332 (34.7)	6173 (34.8)	6159 (34.7)	
Current	3683 (10.4)	1808 (10.2)	1875 (10.6)	
Unknown	240 (0.7)	121 (0.7)	119 (0.7)	
Alcohol status, N (%)				0.077
Never	2364 (6.7)	1251 (7.0)	1113 (6.3)	
Previous	1749 (4.9)	738 (4.2)	1011 (5.7)	
Current	31264 (88.0)	15706 (88.5)	15558 (87.6)	
Unknown	133 (0.4)	60 (0.3)	73 (0.4)	
Moderate activity, yes, N (%)	21152 (59.6)	10515 (59.2)	10637 (59.9)	0.014
Healthy diet, yes, N (%)	7281 (20.5)	3611 (20.3)	3670 (20.7)	0.008
waist circumference, mean (SD), mm	90.67 (13.51)	90.75 (13.52)	90.59 (13.51)	0.012
Weight, mean (SD), kg	78.51 (16.08)	78.60 (16.11)	78.43 (16.05)	0.011
Body mass index, mean (SD), kg/m2	27.55 (4.85)	27.57 (4.87)	27.53 (4.82)	0.008
glucose, mean (SD), mmol/L	5.14 (1.24)	5.15 (1.29)	5.12 (1.18)	0.029
HbA1c, mean (SD), mmol/L	36.21 (6.90)	36.26 (6.96)	36.17 (6.83)	0.012
C-reactive protein, mean (SD), mg/L	2.58 (4.19)	2.63 (4.42)	2.53 (3.94)	0.024
Triglycerides, mean (SD), mmol/L	1.76 (1.02)	1.76 (1.02)	1.76 (1.03)	0.007
High-density lipoprotein cholesterol, mean (SD), mmol/L	1.44 (0.38)	1.44 (0.38)	1.44 (0.38)	0.004
Low-density lipoprotein cholesterol, mean (SD), mmol/L	3.55 (0.88)	3.56 (0.87)	3.54 (0.88)	0.024
lipid lowering medication, N (%)	27215 (76.6)	13593 (76.6)	13622 (76.7)	0.004
Diabetes mellitus				0.071
No	26093 (73.5)	13193 (74.3)	12900 (72.7)	
Yes	9417 (26.5)	4562 (25.7)	4855 (27.8)	
Uric acid, mean (SD), μmol/L	308.87 (80.47)	308.45 (80.25)	309.29 (80.70)	0.01
Vitamin D, mean (SD), nmol/L	48.73 (21.22)	48.81 (21.20)	48.64 (21.24)	0.008

**Table 5:** Longitudinal association of CHD with occurrence of colon and rectal polyp.

	HR (Colon polyp)	HR (Rectal polyp)
After PSM	1.57(1.45-1.69, p<0.001)	1.49(1.33-1.66, p<0.001)

## Discussion

This study examined the potential association between CHD and the risk of new-onset CRPs within the UK Biobank study population. Our findings provide novel insights into the relationship between CHD and the likelihood of developing CRPs. This association remained robust and statistically significant even after conducting subgroup analyses and adjusting for confounding variables. These results are consistent with previous research on various cancers and dementia, emphasizing the significant impact of CHD on the development of diverse health conditions (15, 16), Notably, our results indicated a higher prevalence of CRPs among individuals with CHD. By employing a prospective cohort design, we differentiated our study from prior cross-sectional investigations, providing stronger evidence of the elevated risk of CRPs in patients with CHD (9, 17). Consequently, individuals diagnosed with CHD should remain vigilant regarding the development of CRPs and consider undergoing more frequent colonoscopic examinations.

Both CHD and colorectal cancer are among the leading causes of mortality globally. Numerous studies indicate that these two diseases share similar risk factors and biological characteristics (18, 19). Obesity and cardiometabolic disorders are prevalent overlapping factors between CHD and CRC (19). Our study revealed a higher prevalence of cardiometabolic disorders among patients with CHD, aligning with the established understanding that CHD increases the risk of CRPs. Notably, certain medications frequently employed in the management of cardiovascular disease, such as aspirin and statins, have demonstrated potential in reducing the incidence of CRPs (20, 21). In our analysis, the use of lipid-lowering medications was associated with lower odds and hazard ratios among CHD patients, suggesting a possible inverse relationship between lipid reduction and the formation of CRPs. It is essential to highlight that baseline lipid levels, including triglycerides, high-density lipoprotein (HDL), and low-density lipoprotein (LDL), did not exhibit statistically significant differences, likely attributable to other confounding factors.

Epidemiological evidence suggests that physical activity can diminish the risk of colorectal cancer by up to 20% through mechanisms such as enhancing basal metabolic rate, reducing insulin resistance, and improving immune function (22). Conversely, an unhealthy diet characterized by excessive consumption of processed foods has been associated with an elevated risk of colorectal cancer (23). The intake of red and processed meats correlates with a higher prevalence of cardiovascular events and colorectal cancer (23). Our study is the first to demonstrate that engaging in moderate physical activity and maintaining a healthy diet may significantly reduce the onset of new CRPs in patients with CHD.

Additionally, our study investigated the influence of income on the emergence of CRPs among patients with CHD. We found that higher income levels were associated with a decreased occurrence of CRPs. This finding aligns with epidemiological evidence indicating that colorectal cancer exhibits a lower incidence rate among individuals in higher income brackets (24).Several factors may contribute to this observation; for instance, participants with higher incomes typically have improved access to resources—both temporal and financial—that facilitate the prioritization of health-promoting activities such as physical exercise and a nutritious diet (25).

Numerous studies have established a correlation between DM and CRPs (26–28). The glucose-lowering medication metformin has been associated with a reduced prevalence of polyps, adenomas, and CRC (28). Furthermore, the presence of DM interacts with various colorectal cancer risk factors, including serum urea levels (29, 30). In our study, patients with CHD and DM exhibited the highest OR and HR, indicating that DM significantly heightens the risk of new-onset CRPs within this population.

The strengths of our study include a substantial sample size, a prospective design, an extended follow-up duration, and comprehensive adjustments for multiple confounding variables. However, several limitations merit attention. Firstly, the predominance of participants of European descent may restrict the generalizability of our findings to other ethnic groups. Secondly, the observational nature of the study limits our ability to establish a causal relationship between CHD and the development of CRPs, underscoring the necessity for future randomized clinical trials to elucidate causality. Thirdly, self-reporting may introduce potential biases, a common limitation in population-based studies. Future research should consider alternative methodologies to enhance the accuracy of noise level exposure measurements, although the associated high costs and compliance challenges present obstacles in large cohort studies. Lastly, detection bias remains a pertinent concern; CHD patients, often undergoing antithrombotic therapy, may experience a higher incidence of gastrointestinal bleeding, resulting in more frequent endoscopic evaluations and subsequently elevated rates of asymptomatic polyp detection. Additionally, the enhanced clinical surveillance of CHD patients may lead to more frequent colonoscopies.

Unfortunately, we lacked complete data on colonoscopy procedures for all participants, which hindered our ability to accurately quantify this difference or constrain our analysis to a uniformly screened population, as ideally recommended. Consequently, we cannot dismiss the possibility that the observed association is partially influenced by increased detection within the CHD group. Although the United Kingdom’s population-based bowel cancer screening program standardizes screening invitations by age, and we employed PSM to balance various covariates—including socioeconomic status—residual confounding due to varying examination rates remains a concern. Future investigations that incorporate detailed data on the frequency and indications for endoscopic procedures would be invaluable in further addressing this issue. Additionally, utilizing ICD-10 codes to identify the onset of CRPs may be associated with healthcare utilization, potentially leading to misclassification bias. Nevertheless, our findings align with previous studies, bolstering the reliability of our results.

## Conclusions

Our study presents additional evidence indicating that prolonged exposure to CHD is associated with an elevated risk of the onset of CRPs. We observed that higher income levels, moderate physical activity, healthy dietary practices, and the use of lipid-lowering medications were correlated with a decreased risk of CRPs among patients with CHD. Conversely, the presence of DM emerged as an additional risk factor among this population. These findings suggest that CHD represents an independent risk factor for CRPs, highlighting the necessity for regular screening and lifestyle modifications in patients with CHD to mitigate the risk of colorectal cancer.

## Data Availability

The original contributions presented in the study are included in the article/**Supplementary Material**. Further inquiries can be directed to the corresponding authors.
