# Supplementary material for: The association between coronary heart disease and the risk of developing colorectal polyps: insights from the UK Biobank
Source: Front Oncol. 2025 Nov 5;15:1643394. doi: 10.3389/fonc.2025.1643394 (PMC12626829; doi:10.3389/fonc.2025.1643394)
Supplement: Supplementary file 1 [file Table1.docx]

**Supplementary Table 1: the basic information based on the status of Rectal polyps**

|  | Overall | Rectal polyps (No) | Rectal polyps (Yes) | P value |
| --- | --- | --- | --- | --- |
| **N** | 422200 | 412763 | 9437 |  |
| **Sex, Female (%)** | 192251 (45.5) | 187901 (45.5) | 4350 (46.1) | 0.274 |
| **age (mean (SD))** | 56.51 (8.09) | 56.51 (8.09) | 56.49 (8.11) | 0.871 |
| **ethnic (%)** |  |  |  | 0.163 |
| White | 381662 (90.4) | 373172 (90.4) | 8490 (90.0) |  |
| Mixed | 38246 (9.1) | 37344 (9.0) | 902 (9.6) |  |
| Asian or Asian British | 2292 (0.5) | 2247 (0.5) | 45 (0.5) |  |
| **University or college educational level, N (%)** | 286463 (67.9) | 280019 (67.8) | 6444 (68.3) | 0.367 |
| **employment (%)** |  |  |  | 0.568 |
| Worked | 244849 (58.0) | 239440 (58.0) | 5409 (57.3) |  |
| Retired | 139787 (33.1) | 136605 (33.1) | 3182 (33.7) |  |
| Unemployed | 32796 (7.8) | 32054 (7.8) | 742 (7.9) |  |
| None of the above | 4768 (1.1) | 4664 (1.1) | 104 (1.1) |  |
| **Income, N (%), pounds/year** |  |  |  | <0.001 |
| Unknown | 58802 (14.0) | 57446 (13.9 | 1356(14.4) |  |
| Less than 18,000 | 75566 (17.9) | 73576 (17.8) | 1990 (21.1) |  |
| 18,000 to 30,999 | 90799 (21.5) | 88587 (21.5) | 2212 (23.4) |  |
| 31,000 to 51,999 | 97782 (23.2) | 95656 (23.2) | 2126 (22.5) |  |
| 52,000 to 100,000 | 78235 (18.5) | 76829 (18.6) | 1406 (14.9) |  |
| >100,000 | 21016 (5.0) | 20669 (5.0) | 347 (3.7) |  |
| **Townsend deprivation index, mean (SD)** | -1.36 (3.05) | -1.37 (3.05) | -1.11 (3.15) | <0.001 |
| **Smoking status, N (%)** |  |  |  | 0.334 |
| Never | 230232 (54.5) | 225154 (54.5) | 5078 (53.8) |  |
| Previous | 145104 (34.4) | 141794 (34.4) | 3310 (35.1) |  |
| Current | 44436 (10.5) | 43434 (10.5) | 1002 (10.6) |  |
| Unknown | 2428 ( 0.6) | 2381 ( 0.6) | 47 (0.5) |  |
| **Alcohol status, N (%)** |  |  |  | <0.001 |
| Never | 18357 (4.3) | 18055 (4.4) | 302 (3.2) |  |
| Previous | 13745 (3.3) | 13415 (3.3) | 330 (3.5) |  |
| Current | 388920 (92.1) | 380136 (92.1) | 8784 (93.1) |  |
| Unknown | 1178 (0.3) | 1157 (0.3) | 21 (0.2) |  |
| **Moderate activity, yes, N (%)** | 269485 (63.8) | 263595 (63.9) | 5890 (62.4) | 0.004 |
| **Healthy diet, yes, N (%)** | 86650 (20.5) | 84947 (20.6) | 1703 (18.0) | <0.001 |
| **waist circumference , mean (SD), mm** | 90.29 (13.48) | 90.29 (13.47) | 90.27 (13.66) | 0.87 |
| **Weight, mean (SD), kg** | 78.05 (15.94) | 78.05 (15.94) | 78.07 (16.03) | 0.912 |
| **Body mass index, mean (SD), kg/m2** | 27.43 (4.81) | 27.43 (4.81) | 27.42 (4.78) | 0.785 |
| **glucose, mean (SD), mmol/L** | 5.12 (1.24) | 5.12 (1.24) | 5.13 (1.26) | 0.505 |
| **HbA1c, mean (SD), mmol/L** | 36.12 (6.74) | 36.12 (6.74) | 36.21 (6.77) | 0.211 |
| **C-reactive protein, mean (SD), mg/L** | 2.60 (4.35) | 2.60 (4.35) | 2.58 (4.14) | 0.622 |
| **Triglycerides, mean (SD), mmol/L** | 1.75 (1.03) | 1.75 (1.03) | 1.73 (1.02) | 0.185 |
| **High-density lipoprotein cholesterol, mean (SD), mmol/L** | 1.45 (0.38) | 1.45 (0.38) | 1.45 (0.39) | 0.23 |
| **Low-density lipoprotein cholesterol, mean (SD), mmol/L** | 3.56 (0.87) | 3.56 (0.87) | 3.55 (0.88) | 0.415 |
| **No-lipid lowering medication, N (%)** | 395284 (93.6) | 386557 (93.7) | 8727 (92.5) | <0.001 |
| **CHD** | 17806 (4.2) | 17151 (4.2) | 655 (6.9) | <0.001 |
| **Diabetes mellitus** |  |  |  | <0.001 |
| None | 389826 (92.3) | 381725 (92.5) | 8101 (85.8) |  |
| Yes (during follow-up) | 15601 (3.7) | 14910 (3.6) | 691 (7.3) |  |
| Yes (at basis) | 16773 (4.0) | 16128 (3.9) | 645 (6.8) |  |
| **Uric acid, mean (SD), μmol/L** | 309.08 (80.38) | 309.06 (80.36) | 309.91 (81.31) | 0.31 |
| **Vitamin D, mean (SD), nmol/L** | 48.59 (21.06) | 48.58 (21.06) | 48.92 (21.07) | 0.116 |
