# Supplementary material for: The association between coronary heart disease and the risk of developing colorectal polyps: insights from the UK Biobank
Source: Front Oncol. 2025 Nov 5;15:1643394. doi: 10.3389/fonc.2025.1643394 (PMC12626829; doi:10.3389/fonc.2025.1643394)
Supplement: Supplementary file 2 [file Table2.docx]

Supplementary table 2：Longitudinal association of CHD with risk of rectal polyp based on the group of different categorical variables.

| Dependent: rectal polyp |  | HR (95%CI, P value) | |
| --- | --- | --- | --- |
|  |  | CHD (No) | CHD (Yes) |
| Income | Less than 18,000 | Reference | 1.42(1.22-1.65, p<0.001) |
|  | 18,000 to 30,999 | 0.92(0.87-0.99, p=0.026) | 1.59(1.35-1.87, p<0.001) |
|  | 31,000 to 51,999 | 0.83(0.78-0.90, p<0.001) | 1.46(1.19-1.78, p<0.001) |
|  | 52,000 to 100,000 | 0.69(0.65-0.75, p<0.001) | 0.96(0.69-1.33, p=0.807) |
|  | >100,000 | 0.63(0.56-0.72, p<0.001) | 1.17(0.63-2.17, p=0.621) |
| Moderate activity | No | Reference | 1.69(1.51-1.89, p<0.001) |
|  | Yes | 0.95(0.91-0.99, p=0.023) | 1.57(1.40-1.77, p<0.001) |
| Healthy diet | No | Reference | 1.67(1.53-1.83, p<0.001) |
|  | Yes | 0.84(0.80-0.89, p<0.001) | 1.45(1.44-1.83, p<0.001) |
| medication | Lowing lipid | Reference | 1.00(0.81-1.23, p=0.982) |
|  | Other | 0.81(0.75-0.89, p<0.001) | 1.51(1.34-1.71, p<0.001) |
| Diabetes mellitus | No | Reference | 1.53(1.38-1.69, p<0.001) |
|  | Yes | 2.10(1.87-2.36, p<0.001) | 2.72(2.27-3.27, p<0.001) |
